# Stability of the factorial structure of metabolic syndrome from childhood to adolescence: a 6-year follow-up study

**DOI:** 10.1186/1475-2840-10-81

**Published:** 2011-09-21

**Authors:** Vicente Martínez-Vizcaino, Francisco B Ortega, Montserrat Solera-Martínez, Jonatan R Ruiz, Idoia Labayen, Diva Eensoo, Jaanus Harro, Helle-Mai Loit, Toomas Veidebaum, Michael Sjöström

**Affiliations:** 1 Social and Health Care Research Center, University of Castilla-La Mancha, Cuenca, Spain; 2 Unit for Preventive Nutrition, Department of Biosciences and Nutrition at NOVUM, Karolinska Institutet, Huddinge, Sweden; 3 Department of Medical Physiology, School of Medicine, University of Granada, Granada, Spain; 4 Department of Physical Education and Sport, School of Physical Activity and Sport Sciences. University of Granada, Granada, Spain; 5 Department of Nutrition and Food Science, University of the Basque Country, Vitoria, Spain; 6 Institute of Public Health, Estonian Centre of Behavioral and Health Sciences, University of Tartu, Tartu, Estonia; 7 Department of Psychology, University of Tartu, Estonian Centre of Behavioral and Health Sciences, Tartu, Estonia; 8 Department Chronic Diseases, National Institute for Health Development, Estonian Centre of Behavioral and Health Sciences, Tallinn, Estonia; 9 National Institute for Health Development, Estonian Centre of Behavioral and Health Sciences, Tallinn, Estonia

**Keywords:** Tracking, Metabolic syndrome, Confirmatory factor analysis

## Abstract

**Background:**

Metabolic syndrome (MS) is a clustering of cardiometabolic risk factors that is considered a predictor of cardiovascular disease, type 2 diabetes and mortality. There is no consistent evidence on whether the MS construct works in the same way in different populations and at different stages in life.

**Methods:**

We used confirmatory factor analysis to examine if a single-factor-model including waist circumference, triglycerides/HDL-c, insulin and mean arterial pressure underlies metabolic syndrome from the childhood to adolescence in a 6-years follow-up study in 174 Swedish and 460 Estonian children aged 9 years at baseline. Indeed, we analyze the tracking of a previously validated MS index over this 6-years period.

**Results:**

The estimates of goodness-of-fit for the single-factor-model underlying MS were acceptable both in children and adolescents. The construct stability of a new model including the differences from baseline to the end of the follow-up in the components of the proposed model displayed good fit indexes for the change, supporting the hypothesis of a single factor underlying MS component trends.

**Conclusions:**

A single-factor-model underlying MS is stable across the puberty in both Estonian and Swedish young people. The MS index tracks acceptably from childhood to adolescence.

## Background

Metabolic syndrome (MS) is a clustering of cardiometabolic risk factors, and is considered a predictor of cardiovascular disease, type 2 diabetes and mortality [1]. The definition of MS is controversial, but it generally includes insulin resistance or glucose intolerance, hypertension, dyslipidemia and central obesity [2,3].

A number of studies have examined the relationships among the cardiometabolic risk factors included in the MS definition by using exploratory [4,5] or confirmatory factorial analysis (CFA) [6]. Recent studies using CFA suggested that a single factor underlies the MS in children [7] and adolescents [8], as well as in adults [9].

There is no consistent evidence on whether the MS construct works in the same way in different populations and at different stages in life. Particularly, it would be of interest to know to what extent the clustering of cardiometabolic risk factors as well as the MS construct track from childhood to adolescence. A recent review identified seven studies that investigated the stability of the clustering of cardiometabolic risk factors from childhood to adolescence [10]. Most of the studies focused the analyses only on the tracking of the clustering of cardiometabolic risk factors [11] and a few of them also studied the stability over time of a MS composite risk score [12-14]. However, none of them studied the stability of the factorial structure of the MS. One study focused on the stability from childhood to adolescence of factorial structure of MS [15], and did not observe an adequate fit of the proposed models at baseline; therefore further longitudinal analyses were not possible.

In the present study, we aimed: 1) to validate a proposed [7] single factor model underlying metabolic syndrome in Swedish and Estonian children and adolescents, which included waist circumference, triglycerides-to-high density lipoprotein cholesterol (HDL-c) ratio, insulin, and mean arterial pressure (MAP) (cross-sectional study); 2) to examine, whether there is an underlying single factor for the change in cardiometabolic risk factors (i.e. factorial stability of MS) from childhood to adolescence (6-year follow up study); and 3) to examine the tracking of the proposed MS index [7] from the puberty to the adolescence controlling for relevant confounders.

## Methods

### Study sample and design

**Figure 1:**
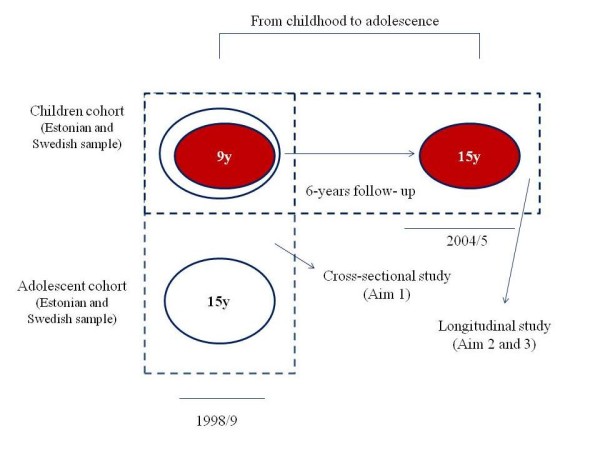
**Study design and aims of the present study**.

Estonian and Swedish participants were originally (in 1998/9, baseline) part of the European Youth Heart Study (EYHS). Study design, selection criteria and sample calculations have been reported elsewhere [16]. In 2004/5 (follow-up), participants were invited to complete the same examination as in 1998/9; the median (percentile 25^th ^- 75^th^) follow-up period was 5.97 years (5.73-6.00). Measurements at baseline and follow-up were made by essentially the same group of trained investigators, and by using the same protocols, which allows performing combined analyses. The follow-up assessment in the Estonian cohort was carried out as part of the longitudinal Estonian Children Personality Behaviour and Health Study [17]. The study design is graphically depicted in Figure 1.

For the study purposes, participants with complete and valid data at baseline on waist circumference, triglycerides, HDL-c, insulin and systolic and diastolic blood pressure were included in the cross-sectional analyses (Aim 1, baseline examination 1998/9): N = 1158 Estonian children (9 year-old, N = 565) and adolescents (15 year-old, N = 593), and N = 906 Swedish children (9 year-old, N = 440) and adolescents (15 year-old, N = 466). Six years later, the children cohort was invited to participate in the follow-up examination. A total of 460 Estonian (dropout rate = 19%) and 174 Swedish (dropout rate = 60%) children with valid data on all the variables mentioned above were included in the study (Aim 2 and 3). There were no significant difference between participants and non-participants in the follow-up studies regarding the main study outcomes, i.e. waist circumference, triglycerides, HDL-c, insulin and blood pressure (all p > 0.1). Higher maternal education was however associated to higher participation rate at follow-up (p < 0.001).

The study protocol was performed in accordance with the ethical standards laid down in the 1961 Declaration of Helsinki (as revised in 2000), and approved by the Research Ethics Committees of University of Tartu (no. 49/30-199), Örebro County Council (no. 690/98) and Huddinge University Hospital (no. 474/98). Children and adolescents gave verbal assent after procedures were explained and one parent or legal guardian provided written informed consent.

#### Central body fat

Waist circumference was taken with a metal anthropometric tape midway between the lower rib margin and the iliac crest, at the end of gentle expiration in triplicate, and the mean was scored [18]. Waist circumference was used as proxy of central adiposity.

#### Blood parameters

Blood samples were taken by venipuncture after an overnight fast. The fasting state was verbally confirmed by the participants before sampling. HDL-c, triglycerides, glucose and insulin levels were measured as reported elsewhere [19].

#### Blood pressure

Systolic and diastolic blood pressure was measured with an automatic oscillometric method (Dinamap model XL critikron, Imc., Tampa, Florida) using standard procedures described elsewhere [20]. The mean arterial pressure (MAP) was calculated as follows: diastolic pressure + [0.333 × (systolic blood pressure - diastolic pressure)].

*Metabolic syndrome index*. We constructed a metabolic syndrome index as the sum of the standardized scores of the four variables that comprised our proposed model multiplied by the factor loadings in the model described in Spanish children [7].

#### Confounders

Several variables potentially related to cardiometabolic risk factors were taken into account. Socioeconomic status was assessed via questionnaire and defined by the *maternal educational level*, coded as 0 (below university education) and 1 (university education). *Pubertal stage *was assessed by a trained researcher by direct observation and according to Tanner and Whitehouse [21]. *Birth weight *data were collected from parental recall. The validity of parents-reported birth weight data has been previously verified in a randomly selected subset of the Swedish-EYHS [22].

*Cardiorespiratory fitness *(VO_2 _ml/kg/min) was estimated by an incremental maximal cycle-ergometer test (Monark 829E Ergomedic, Vansbro, Sweden) as detailed elsewhere [23].

### Statistical analysis

Triglyceride-to-HDL-c ratio and insulin were log transformed in the two samples to adhere to the normality assumptions. Two main set of analyses were conducted on 1) cross-sectional data (Aim 1), and 2) longitudinal data (Aims 2 and 3).

#### Aim 1

The first step was to validate the single factor model underlying MS suggested [7] in the present sample of Estonian and Swedish youth. All the variables were z transformed (mean = 0 and SD = 1) by age and sex. CFA was used to calculate the factor loadings of the cardiometabolic risk factors in the main model, as well as by age and gender groups. Factors loadings are interpreted as correlation coefficients, and measure the strength of the association between the model variables and their overall underlying factor, which is the MS construct itself. Factor loadings were estimated by maximum likelihood methods with the IBM SPSS Amos 19.0. The analyses were performed separately by country, sex and age group (9/15 years). Chi-squared tests were used to examine differences in factors loadings between country, sex, and age groups.

To assess each model's goodness-of-fit to the observed data, we used the chi-squared test, the comparative fit index (CFI), and the standardized root mean square residual (SRMR). Given that the study sample size was relatively large, it is relatively easy for the chi-squared test to show a significant (p < 0.05) lack of fit, so that the results of this test cannot be assessed in isolation. A model was deemed to have a good fit when the CFI was > 0.96 and the SRMR was < 0.08.

#### Aim 2

The stability of the factorial structure of the proposed model was assessed in two ways: (i) estimating CFA indexes of goodness of fit in the 1998/9 cohort (at baseline), and in the 2004/5 cohort (6-year follow up); and (ii) estimating CFA indexes of goodness of fit of a model including the changes from 1998/9 to 2004/5 in waist circumference, triglyceride-to-HDL-c ratio, insulin and MAP.

#### Aim 3

Pearson correlation was used to examine the tracking (stability) of the study cardiometabolic risk factors (waist circumference, log triglyceride-to-HDL-c ratio, log insulin, and MAP), as well as the MS index from childhood (baseline 1998/9) to adolescence (follow up 2004/5). A correlation ranging from 0.25 to 0.50 indicates a fair relationship, a correlation from 0.50 to 0.75 indicates a moderate to good relationship, and a correlation > 0.75 indicates a very good relationship [24]. The stability was also estimated through the intraclass correlation coefficient. Analyses were performed by country and sex. The predictive validity of the persistence in the upper quartile of cardiometabolic risk factors and MS index in the same period time was studied by non conditional binary logistic regression models. Odds ratios were estimated with and without controlling for birth weight, maternal education, and changes 1998-2004 in both Tanner stage and cardiorespiratory fitness.

## Results

Participant characteristics of the children and adolescents have been described elsewhere [25]. The children participating in the longitudinal study were at early puberty at baseline (80.7% for Tanner stage I) and at late puberty six years later at follow-up (69% at Tanner stage 4 or 5).

### Factorial structure of the proposed model (Aim 1)

**Figure 2:**
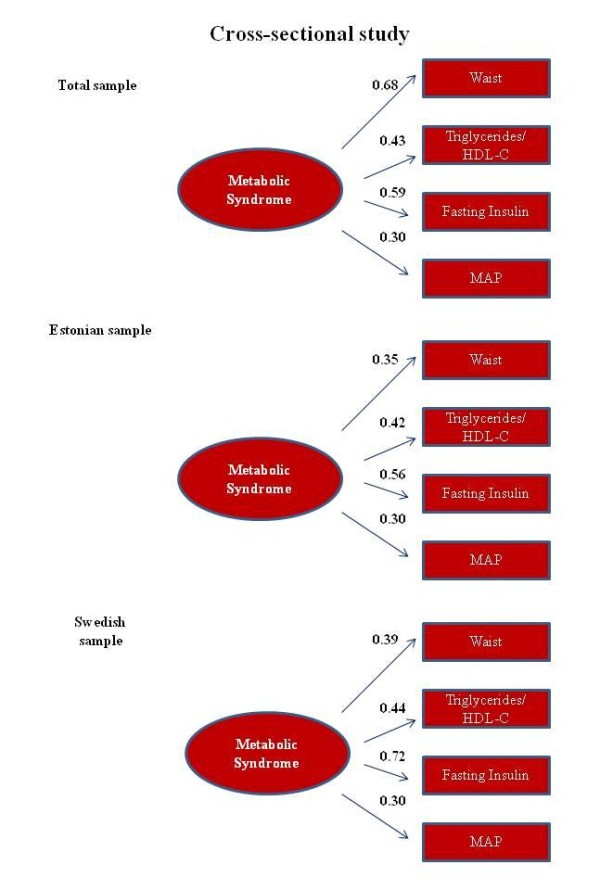
**Factor loading and goodness-of-fit indexes of a single factor models for the metabolic syndrome**. Total sample: n = 2064; χ^2 ^= 10.87, df = 2, p = 0.004; CFI = 0.97; and SRMR = 0.019. Estonian sample: n = 1158; χ^2 ^= 3.70, df = 2, p = 0.159; CFI = 0.99; and SRMR = 0.016. Swedish sample: n = 906; χ^2 ^= 37.4, df = 2, p = 0.001; CFI = 0.96; and SRMR = 0.051. MAP: mean arterial pressure.

The estimates of goodness of fit for the single factor CFA model proposed in the present study for the total sample were adequate (Figure 2). The overall estimates of factor loadings were 0.36, 0.43, 0.59, and 0.30 for waist circumference, triglyceride-to-HDL-c ratio, insulin and MAP, respectively. All standardized fit indices suggested high goodness of fit of the proposed factor structure and supported the MS measurement model (χ^2 ^= 10.87, df = 2, p = 0.004, CFI = 0.97, and SRMR = 0.0198). The goodness of fit indices for the Estonian and Swedish cohort were as follow: χ^2 ^= 3.70, df = 2, p = 0.159; CFI = 0.99; and SRMR = 0.016; and χ^2 ^= 37.4, df = 2, p = 0.001; CFI = 0.96; and SRMR = 0.05, respectively. There were no differences in the MS structure between countries (χ^2^diff = 1.2, df = 3, p = 0.753). Similarly, there were no differences between boys and girls (χ^2^diff = 2.0, df = 3, p = 0.572), or between children and adolescents (χ^2^diff = 6.9, df = 3, p = 0.075) (Additional file 1).

### Stability of the model from puberty to adolescence (Aim 2)

**Figure 3:**
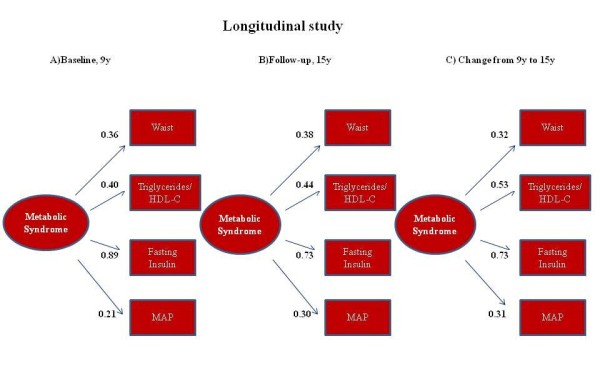
**Goodness of fit and stability of the MetS factorial structure (A) at baseline, (B) after 6 yr follow up, and (C) including as components the change in the variables from 1998 to 2005**. A: n = 634; χ^2 ^= 5.6, df = 2, p = 0.06; CFI = 0.98; and SRMR = 0.026. B: n = 634; χ^2 ^= 6.7, df = 2, p = 0.03; CFI = 0.97; and SRMR = 0.027. C: n = 634 χ^2 ^= 0.32, df = 2, p = 0.85; CFI = 0.1; and SRMR = 0.006. MAP: mean arterial pressure.

Figure 3 depicts the analysis of the stability of the factorial structure of MS in the participants included in the follow up study. The estimates of goodness of fit for the one-factor CFA model displayed good fit both at baseline and after six years follow up. We also assessed the stability of the construct testing a model including the differences between 1998/9 and 2004/5 in the studied cardiometabolic risk factors (i.e. waist circumference, triglyceride-to-HDL-c ratio, insulin and MAP). This one-factor model displayed good fit indexes for the change, supporting the hypothesis that a single factor underlying trends in MS components.

### Tracking analyses

Pearson correlation coefficients showing the association between cardiometabolic risk factors at baseline and at follow-up were moderate to high except for insulin that was low (Table 1). Correlations coefficients were higher for waist circumference. Overall, no remarkable differences by sex or by country were found. The strength of the relationship between values for MS index from 1998 to 2004 was also moderate.

**Table 1:** Pearson correlation coefficients between cardiometabolic risk variables and metabolic syndrome index from childhood (9 year-old) to adolescence (15 year-old) by gender and by country

Variable	Estonia		Sweden		Total	
	Boys (n = 222)	Girls (n = 261)	Boys (n = 130)	Girls (n = 148)	Boys (n = 352)	Girls (n = 409)
Waist	0.683	0.729	0.746	0.602	0.685	0.669
log TG/HDL-c	0.360	0.368	0.472	0.392	0.381	0.375
log insulin	0.221	0.142	0.287	0.202	0.318	0.227
MAP	0.418	0.361	0.410	0.452	0.400	0.398
MS index	0.389	0.399	0.535	0.308	0.431	0.379

The European Youth Heart Study, 1998-2004.

Abbreviations: log TG/HDL: log triglyceride-to-HDL-cholesterol ratio; MAP: mean arterial pressure; MS: metabolic syndrome.

The concordance between childhood and adolescence of the z scores of the cardiometabolic risk variables measured by the intraclass correlation coefficient showed a similar pattern to the inter-age correlation coefficients (Additional file 2). The 1998-2004 concordance of the values of the MS index was moderate, and similar to the variables that included in the index.

Table 2 presents the odds ratios for persistence in the upper quartile of cardiometabolic risk factors and MS index from childhood to adolescence in Estonia and Sweden. The pattern of predictability was similar in both, Estonian and Swedish samples. The risk of remaining in the upper quartile of MS index from childhood to adolescence was twofold compared to children remaining in the other categories.

**Table 2:** Odds ratio for persistence in the upper quartile of cardiometabolic risk factors and metabolic syndrome index from childhood (9 year-old) to adolescence (15 year-old) by country

Variable^a^	Estonia (n = 483)		Sweden (n = 278)		Total (n = 761)	
	OR	95% CI	OR	95% CI	OR	95% CI
Waist	9.84	6.12-15.83	13.58	7.10-25.96	11.04	7.53-16.19
log TG/HDL-c	3.27	2.07-5.18	4.35	2.19-8.66	2.57	2.44-5.23
log insulin	1.70	1.07-2.70	2.55	1.22-5.33	1.90	1.28-2.81
MAP	3.81	2.43-5.96	2.89	1.60-5.20	3.44	2.41-4.91
MS index	2.03	1.28-3.21	2.64	1.25-5.57	2.18	1.47-3.23
Adjusted^b^						
Waist	9.32	5.51-15.76	16.08	6.82-37.92	10.93	6.99-17.10
log TG/HDL-c	3.32	2.02-5.43	5.89	2.21-14.45	3.82	2.48-5.89
log insulin	1.53	0.93-2.50	2.69	1.10-6.59	1.74	1.13-2.68
MAP	3.80	2.36-6.05	2.55	1.29-5.05	3.40	2.30-5.07
MS index	1,98	1.21-3.25	3.33	1.33-8.36	2.24	1.45-3.45

The European Youth Heart Study, 1998-2004.

^a ^Standardized values for gender and age.

^b ^Controlling for: maternal education, change 98-04 in Tanner stage, change 98-04 in cardiorespiratory fitness, and birth weight

Abbreviations: log TG/HDL: log triglyceride-to-HDL-cholesterol ratio; MAP: mean arterial pressure; MS: metabolic syndrome.

## Discussion

Three main findings are derived from this study. First, the present data support the validity of a single factor model underlying MS in Estonian and Swedish children and adolescent, as it was previously observed in Spanish children [7]. Second, the model structure was stable from childhood to adolescence, i.e. the changes from 9 y to 15 y in the model's components fitted as a single factor. Third, we found that the cardiometabolic risk factors clustering and the MS index itself showed a good tracking and predictability from childhood to adolescence.

Several studies have examined the relationship among the several traits included on MS using exploratory factor analysis. The number of described factors underlying MS ranges from 1 to 4 [4,9], possibly due to the proper features of this type of analysis [9]. In our previous study conducted in Spanish children [7], we observed one single factor for the model including waist circumference, triglyceride-to-HDL-c ratio, insulin and MAP. The present study confirms the structural validity of this one-factor model in all the groups studied, i.e. children and adolescents, boys and girls and Swedish and Estonian.

Goodman et al, using data from the Fels Longitudinal Study [15] investigated the stability of factor structure of MS across pubertal development using CFA, but its data did not support the hypothesis neither that a single factor nor multiple factors underlying MS already at baseline, not allowing therefore further longitudinal analyses. Two complementary analyses support the stability of MS as a single factor model from childhood to adolescence: first, no significant differences were found between the factor loadings in childhood and adolescence, and second, a model including the differences on each variable before and after the puberty also fits as a single factor.

There is consistent evidence about the tracking of every cardiometabolic risk factor integrating MS. However, the number of studies focused to examine the stability and predictability of cardiometabolic risk factor clustering is limited [10], and only a few have analyzed the tracking of MS as a composite quantitative index [12,14]. Our study confirmed the stability of cardiometabolic risk factors across the puberty, though the magnitude of this stability differs substantially among the different variables.

A joint statement from the American Diabetes Association and the European Association for the Study of Diabetes recommends expanding the evidence regarding the use of scales based on continuous variables to measure metabolic risk [26]. In the same way, a WHO Expert Consultation Group [27] has pointed out as limitation for the clinical usefulness of the construct MS the dichotomization of its diagnosis and the risk factors to define MS. Furthermore, the summative scales proposed until now assume that each factor has associated the same risk, and there is no evidence to support this assumption. The MS index is an outcome of the single factor model fitting, and have been validated previously [7]. The findings from the present study strengthen the validity of the MS index by showing acceptable indexes of tracking from childhood to adolescence.

The present study has several limitations. The dropout rates in the Swedish sample were high. However, the construct validity of a model using CFA does not require representativeness of the study sample. In addition, the sample size used in the longitudinal analyses was far larger than needed for CFA [28].

Secondly, most of our analyses have been performed in a mixed Swedish and Estonian sample, and we are conscious that no objective analysis of MS it's possible without connecting it to a well-defined population or ethnicity; for example, differences in the prevalence of cardiometabolic risk factors between descent of Turkish and Moroccan have been described in Dutch cohort of overweight/obesity children [29]. In the same way, increased triglyceride and low HDL-c levels have been found the most frequent components of MS in Qatari children [30], or surprisingly a follow up study in the Seychelles have described that elevated total cholesterol tended to decreased whereas the prevalence of MS increased significantly [31]. However, this concern may be not too relevant in our study in light of when we analyzed the factorial structure of MS separately in the Swedish and Estonian samples the coefficients were very similar in both models (Figure 2).

Theoretically, when a factorial model of MS is assessed by CFA, a specific model has to be chosen out of a number of alternative models. Our single factor model is based on an earlier validation study, and its components are risk factors included in most of the current diagnosis criteria of MS.

We believe that the results of the present study have important clinical implications, due to the fact that it supports the hypothesis that MS is a single pathogenic entity. It provides evidence about the more efficient strategies for prevention and control of MS may be those (i.e. promotion physical activity) that influence the whole clustering of risk factors; therefore, do not support strategies focused on individualized approaches of each risk factor component of MS, as has been proposed [15]. Moreover, and considering the stability of the cardiometabolic risk factors levels across the puberty, the implementation of the prevention activities may start at childhood.

## Conclusions

In conclusion, the results from the present study suggest that a single factor model underlies MS in Estonian and Swedish children and adolescents and that this model structure is stable from childhood to adolescence. The clustering of cardiometabolic risk factors included in the model, as well as the MS index itself, showed acceptable indexes of tracking and predictability.

## List Of Abbreviations

MS: metabolic syndrome; CFA: confirmatory factor analysis; HDL-c: high-density lipoprotein cholesterol; MAP: mean arterial pressure; CFI: comparative fit index; SRMR: standardized root mean square residual;

## Competing interests

The authors declare that they have no competing interests.

## Authors' contributions

MS and TV were responsible for the study design and data collection. MSM contributed to the data analyses and review/edited the manuscript. VMV contributed to the data analyses and drafted the manuscript. FBO, JRR, and IL contributed to the data analysis, the discussion and reviewed/edited the manuscript. DE, JH, HL, TV and MS contributed to the discussion and reviewed/edited the manuscript. All authors read and approved the final manuscript.

## Supplementary Material

Additional file 1 **Factor loading and goodness-of-fit indexes for the single-factor model for the metabolic syndrome, by sex and age groups**. Girls: n = 1087; χ2 = 3.9, df = 2, p = 0.142; CFI = 0.99; and SRMR = 0.016. Boys: n = 977; χ2 = 13.4, df = 2, p = 0.001; CFI = 0.93; and SRMR = 0.033. Nine years: n = 1005; χ2 = 13.0, df = 2, p = 0.002; CFI = 0.95; and SRMR = 0.030. Fifteen years: n = 1059; χ2 = 16.4, df = 2, p = 0.001; CFI = 0.91; and SRMR = 0.032. MAP: mean arterial pressure. Goodness-of-fit indexes for the single-factor model for the metabolic syndrome, by sex and age groups. Click here for file

Additional file 2 **Intraclass correlation coefficients between gender and age standardized cardiometabolic risk variables and metabolic syndrome index from childhood (9 year-old) to adolescence (15 year-old), by country**. The European Youth Heart Study, 1998-2004. Concordance between childhood and adolescence of the z scores of the cardiometabolic risk variables measured by the intraclass correlation coefficient. Click here for file

## References

[B1] Eberly LE Prineas R Cohen JD Vazquez G Zhi X Neaton JD Kuller LH Multiple Risk Factor Intervention Trial Research G Metabolic syndrome: risk factor distribution and 18-year mortality in the multiple risk factor intervention trial Diabetes Care 2006 29 1 123 130 10.2337/diacare.29.01.06.dc05-1320 16373907

[B2] Grundy SM Cleeman JI Daniels SR Donato KA Eckel RH Franklin BA Gordon DJ Krauss RM Savage PJ Smith SC Jr Diagnosis and Management of the Metabolic Syndrome: An American Heart Association/National Heart, Lung, and Blood Institute Scientific Statement Circulation 2005 112 17 2735 2752 10.1161/CIRCULATIONAHA.105.169404 16157765

[B3] Sangun O Dündar B Köşker M Pirgon O Dündar N Prevalence of Metabolic Syndrome in Obese Children and Adolescents using Three Different Criteria and Evaluation of Risk Factors J Clin Res Pediatr Endocrinol 2011 3 2 70 76 10.4274/jcrpe.v3i2.15 21750635 PMC3119444

[B4] Earl SF Chaoyang L Defining the Metabolic Syndrome in Children and Adolescents: Will the Real Definition Please Stand Up? The Journal of pediatrics 2008 152 2 160 164 e113 10.1016/j.jpeds.2007.07.056 18206681

[B5] Shen BJ Goldberg RB Llabre MM Schneiderman N Is the factor structure of the metabolic syndrome comparable between men and women and across three ethnic groups: the Miami Community Health Study Ann Epidemiol 2006 16 2 131 137 10.1016/j.annepidem.2005.06.049 16257230

[B6] Shen BJ Todaro JF Niaura R McCaffery JM Zhang J Spiro A Ward KD Are metabolic risk factors one unified syndrome? Modeling the structure of the metabolic syndrome X Am J Epidemiol 2003 157 8 701 711 10.1093/aje/kwg045 12697574

[B7] Martinez-Vizcaino V Martinez MS Aguilar FS Martinez SS Gutierrez RF Lopez MS Martinez PM Rodriguez-Artalejo F Validity of a single-factor model underlying the metabolic syndrome in children: a confirmatory factor analysis Diabetes Care 2010 33 6 1370 1372 10.2337/dc09-2049 20299487 PMC2875456

[B8] Li C Ford ES Is there a single underlying factor for the metabolic syndrome in adolescents? A confirmatory factor analysis Diabetes Care 2007 30 6 1556 1561 10.2337/dc06-2481 17363752

[B9] Pladevall M Singal B Williams LK Brotons C Guyer H Sadurni J Falces C Serrano-Rios M Gabriel R Shaw JE A single factor underlies the metabolic syndrome: a confirmatory factor analysis Diabetes Care 2006 29 1 113 122 10.2337/diacare.29.01.06.dc05-0862 16373906

[B10] Camhi SM Katzmarzyk PT Tracking of cardiometabolic risk factor clustering from childhood to adulthood International Journal of Pediatric Obesity 5 2 122 129 10.3109/17477160903111763 19593726

[B11] Chen W Srinivasan SR Li S Xu J Berenson GS Clustering of Long-term Trends in Metabolic Syndrome Variables from Childhood to Adulthood in Blacks and Whites American Journal of Epidemiology 2007 166 5 527 533 10.1093/aje/kwm105 17573336

[B12] Eisenmann JC Welk GJ Wickel EE Blair SN Stability of variables associated with the metabolic syndrome from adolescence to adulthood: The Aerobics Center Longitudinal Study American Journal of Human Biology 2004 16 6 690 696 10.1002/ajhb.20079 15495227

[B13] Bao W Srinivasan SR Wattigney WA Berenson GS Persistence of Multiple Cardiovascular Risk Clustering Related to Syndrome × From Childhood to Young Adulthood: The Bogalusa Heart Study Arch Intern Med 1994 154 16 1842 1847 10.1001/archinte.154.16.1842 8053753

[B14] Katzmarzyk PT Perusse L Malina RM Bergeron J Despres JP Bouchard C Stability of indicators of the metabolic syndrome from childhood and adolescence to young adulthood: the Quebec Family Study J Clin Epidemiol 2001 54 2 190 195 10.1016/S0895-4356(00)00315-2 11166535

[B15] Goodman E Li C Tu YK Ford E Sun SS Huang TT Stability of the factor structure of the metabolic syndrome across pubertal development: confirmatory factor analyses of three alternative models J Pediatr 2009 155 3 S5 e1 8 10.1016/j.jpeds.2009.04.045 PMC3763727 19732562

[B16] Wennlof AH Yngve A Sjostrom M Sampling procedure, participation rates and representativeness in the Swedish part of the European Youth Heart Study (EYHS) Public Health Nutr 2003 6 3 291 299 12740078 10.1079/PHN2002425

[B17] Harro J Merenakk L Nordquist N Konstabel K Comasco E Oreland L Personality and the serotonin transporter gene: Associations in a longitudinal population-based study Biol Psychol 2009 81 1 9 13 10.1016/j.biopsycho.2009.01.001 19437595

[B18] Ortega FB Ruiz JR Sjostrom M Physical activity, overweight and central adiposity in Swedish children and adolescents: the European Youth Heart Study Int J Behav Nutr Phys Act 2007 4 1 61 10.1186/1479-5868-4-61 18021444 PMC2211506

[B19] Wennlof AH Yngve A Nilsson TK Sjostrom M Serum lipids, glucose and insulin levels in healthy schoolchildren aged 9 and 15 years from Central Sweden: reference values in relation to biological, social and lifestyle factors Scand J Clin Lab Invest 2005 65 1 65 76 10.1080/00365510410003110 15859028

[B20] Ruiz JR Ortega FB Loit HM Veidebaum T Sjostrom M Body fat is associated with blood pressure in school-aged girls with low cardiorespiratory fitness: the European Youth Heart Study J Hypertens 2007 25 10 2027 2034 10.1097/HJH.0b013e328277597f 17885544

[B21] Tanner JM Whitehouse RH Clinical longitudinal standards for height, weight, height velocity, weight velocity, and stages of puberty Archives of Disease in Childhood 1976 51 3 170 179 10.1136/adc.51.3.170 952550 PMC1545912

[B22] Labayen I Ortega FB Sjostrom M Ruiz JR Early Life Origins of Low-Grade Inflammation and Atherosclerosis Risk in Children and Adolescents J Pediatr 2009 155 5 673 677 10.1016/j.jpeds.2009.04.056 19595364

[B23] Hansen HS Froberg K Nielsen JR Hyldebrandt N A new approach to assessing maximal aerobic power in children: the Odense School Child Study Eur J Appl Physiol 1989 58 6 618 624 10.1007/BF00418508 2731531

[B24] Colton T Statistics in medicine 1974 Boston: Little Brown

[B25] Rizzo NS Ruiz JR Hurtig-Wennlof A Ortega FB Sjostrom M Relationship of Physical Activity, Fitness, and Fatness with Clustered Metabolic Risk in Children and Adolescents: The European Youth Heart Study The Journal of pediatrics 2007 150 4 388 394 10.1016/j.jpeds.2006.12.039 17382116

[B26] Buse JB Ginsberg HN Bakris GL Clark NG Costa F Eckel R Fonseca V Gerstein HC Grundy S Nesto RW Primary Prevention of Cardiovascular Diseases in People With Diabetes Mellitus: A Scientific Statement From the American Heart Association and the American Diabetes Association Circulation 2007 115 1 114 126 17192512 10.1161/CIRCULATIONAHA.106.179294

[B27] Simmons R Alberti K Gale E Colagiuri S Tuomilehto J Qiao Q Ramachandran A Tajima N Brajkovich Mirchov I Ben-Nakhi A The metabolic syndrome: useful concept or clinical tool? Report of a WHO Expert Consultation Diabetologia 2010 53 4 600 605 10.1007/s00125-009-1620-4 20012011

[B28] Kline RB Principles and Practice of Structural Equation Modeling 2005 2 New York: The Guilford Press

[B29] van Vliet M von Rosenstiel IA Schindhelm RK Brandjes DP Beijnen JH Diamant M Ethnic differences in cardiometabolic risk profile in an overweight/obese paediatric cohort in the Netherlands: a cross-sectional study Cardiovasc Diabetol 2009 19 8 2 10.1186/1475-2840-8-2 PMC2642775 19152682

[B30] Rizk N Amin M Yousef M A pilot study on metabolic syndrome and its associated features among Qatari schoolchildren Int J Gen Med 2011 4 521 525 21845059 10.2147/IJGM.S21103 PMC3150174

[B31] Bovet P Romain S Shamlaye C Mendis S Darioli R Riesen W Tappy L Paccaud F Divergent fifteen-year trends in traditional and cardiometabolic risk factors of cardiovascular diseases in the Seychelles Cardiovasc Diabetol 2009 8 34 10.1186/1475-2840-8-34 19558646 PMC2719584

