# Supplementary figures and images for: Stability of the factorial structure of metabolic syndrome from childhood to adolescence: a 6-year follow-up study
Source: Cardiovasc Diabetol. 2011 Sep 21;10:81. doi: 10.1186/1475-2840-10-81 (PMC3193025; doi:10.1186/1475-2840-10-81)

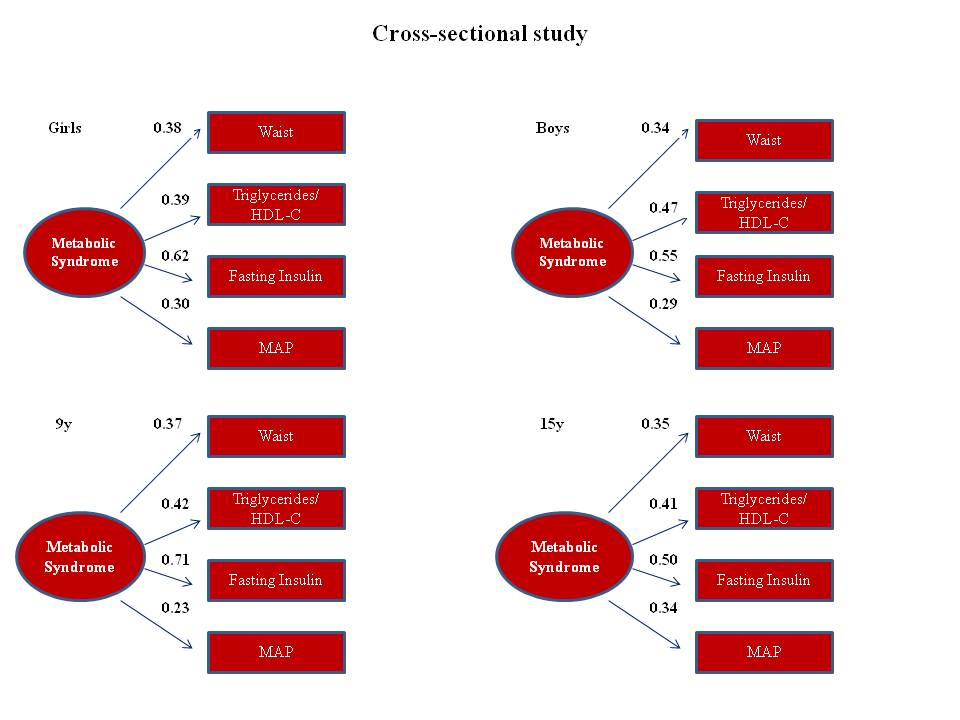

Supplement: Additional file 1 — Factor loading and goodness-of-fit indexes for the single-factor model for the metabolic syndrome, by sex and age groups. Girls: n = 1087; χ2 = 3.9, df = 2, p = 0.142; CFI = 0.99; and SRMR = 0.016. Boys: n = 977; χ2 = 13.4, df = 2, p = 0.001; CFI = 0.93; and SRMR = 0.033. Nine years: n = 1005; χ2 = 13.0, df = 2, p = 0.002; CFI = 0.95; and SRMR = 0.030. Fifteen years: n = 1059; χ2 = 16.4, df = 2, p = 0.001; CFI = 0.91; and SRMR = 0.032. MAP: mean arterial pressure. Goodness-of-fit indexes for the single-factor model for the metabolic syndrome, by sex and age groups. [file 1475-2840-10-81-S1.DOC]
