# Supplementary material for: Stability of the factorial structure of metabolic syndrome from childhood to adolescence: a 6-year follow-up study
Source: Cardiovasc Diabetol. 2011 Sep 21;10:81. doi: 10.1186/1475-2840-10-81 (PMC3193025; doi:10.1186/1475-2840-10-81)
Supplement: Additional file 2 — Intraclass correlation coefficients between gender and age standardized cardiometabolic risk variables and metabolic syndrome index from childhood (9 year-old) to adolescence (15 year-old), by country. The European Youth Heart Study, 1998-2004. Concordance between childhood and adolescence of the z scores of the cardiometabolic risk variables measured by the intraclass correlation coefficient. [file 1475-2840-10-81-S2.DOC]

**Additional file 2.** Intraclass correlation coefficients between gender and age standardized cardiometabolic risk variables and metabolic syndrome index from childhood (9 year-old) to adolescence (15 year-old), by country. The European Youth Heart Study, 1998-2004.

| Variable^a^ | Estonia (n= 483) | | Sweden (n=278 ) | | Total (n=761) | |
| --- | --- | --- | --- | --- | --- | --- |
|  | ICC | 95% CI | ICC | 95% CI | ICC | 95% CI |
| Waist | 0.698  0.370  0.178  0.385  0,394 | 0.649-0.741 | 0.637  0.420  0.238  0.456  0,428 | 0.561-0.702 | 0.680 | 0.639-0.716 |
| log TG/HDL‑c |  | 0.289-0.447 |  | 0.302-0.525 | 0.355 | 0.287-0.419 |
| log insulin |  | 0.088-0.265 |  | 0.174-0.541 | 0.278 | 0.205-0.348 |
| MAP |  | 0.306-0.458 |  | 0.356-0.546 | 0.391 | 0.329-0.350 |
| MS index |  | 0.314-0.486 |  | 0.299-0.542 | 0.402 | 0.335-0.465 |

^a^ Standardized values for gender and age.

Abbreviations: ICC: intraclass correlation coefficient; log TG/HDL: log triglyceride/HDL cholesterol; MAP: mean arterial pressure; MS: metabolic syndrome.
